# Minds, motherboards, and money: futurism and realism in the neuroethics of BCI technologies

**DOI:** 10.3389/fnsys.2014.00086

**Published:** 2014-05-15

**Authors:** Mark A. Attiah, Martha J. Farah

**Affiliations:** ^1^Perelman School of Medicine, University of PennsylvaniaPhiladelphia, PA, USA; ^2^Center for Neuroscience & Society, University of PennsylvaniaPhiladelphia, PA, USA; ^3^Department of Psychology, University of PennsylvaniaPhiladelphia, PA, USA

**Keywords:** neuroethics, brain computer interface (BCI), transhumanism, implants, experimental, enhancement

## Introduction

Brain computer interfaces (BCIs) are systems that enable the brain to send and receive information to and from a computer, bypassing the body's own efferent and afferent pathways. BCIs have been used in experimental animal models to augment perception, motor control and even memory (Velliste et al., 2008; Berger et al., 2011; Torab et al., 2011). Human BCIs include cochlear implants and a host of experimental devices including retinal implants (Niparko et al., 2010; Klauke et al., 2011). BCI technology holds the potential to benefit humanity greatly, but also the potential to do harm, and its ethical implications have therefore been addressed by a number of commentators.

## The problem with science fiction

Commonly addressed ethical challenges with BCIs concern a future when the technology is used to give people superhuman abilities, or to control their thoughts and desires. The idea of human beings with computer-augmented brains engenders a wide range of reactions from laypersons, mostly negative (Lipsman et al., 2009; Lebedev, 2014). There is revulsion toward these so-called cyborgs occupying the “uncanny valley” between fellow human and robot (Mori, 1970). Some fear being overtaken by cyborgs with superior perceptual, cognitive and motoric powers while others are afraid that BCIs imposed upon us would result in mind control. Still others foresee tremendous benefit for humanity, with BCI enhancements elevating individuals and society.

Positive or negative, these images of computer-augmented brains are as speculative as they are vivid. Therein lie two significant challenges for ethical analysis of BCIs. First, we have strong emotional reactions to the prospects of humans with cyborg brains. Second, our limited real-world experience with BCIs deprives our ethical analyses of a foundation of pragmatic empirical knowledge. As a result, our perspectives on the ethical and societal impact of BCIs are based primarily on gut reactions, either “the wisdom of repugnance” (as exemplified by Francis Fukuyama's nomination of transhumanism as “The World's Most Dangerous Idea, 2004”) or the blind faith that technology can fix and improve everything (as exemplified by the mission statement of Humanity+, which promotes “the ethical use of technology to expand human capacities… [to make them] better than well” (http://humanityplus.org/).

In this essay, we describe an approach to the ethical analysis of BCIs that resists the emotional pull of futuristic scenarios without denying the importance of the questions they raise. In so doing, we address the more prosaic ethical issues that confront us now, which are too often overshadowed by the more sensational prospects of cyborgs, mind control and transhumanism. Finally, we indicate pathways through which seemingly fantastical futuristic scenarios can be more knowledgeably anticipated and addressed.

## Three eras of BCIs and their ethical challenges

As an organizing framework, we will distinguish among three different eras of BCI development, near-term, medium-term, and long-term, and distinguish among the different ethical issues associated with each.

### Long-term challenges

The futuristic ethical concerns already mentioned arise in the long-term future. We define this era as when BCIs are routinely used to augment human brains, giving users perceptual, cognitive and motoric abilities that may greatly exceed those of the unenhanced, and transforming emotional life as well. At this point, cyborgs will differ radically from unenhanced humans. One concern is that they will view us as a different, and inferior, life form, much as we now regard chimpanzees (Warwick, 2003). Loss of individuality is another long-term future scenario. If BCIs are used for direct brain-to-brain contact, this would enable new modes of communication but also lead to the possible replacement of individual mental lives by a network in which individual brains are mere nodes (Warwick, 2003).

Thoughts, feelings and memories that can be stored and shared as bits of data will not degrade as readily as human brain tissue. While other prosthetics such as artificial hips or heart valves can replace failing bodily structures, BCIs offer the first possibility of increased longevity of a person's consciousness. Immortality may even be possible (McGee and Maguire, 2007).

These BCI scenarios lead to interesting questions about what it means to be human. Would we be human if we could see infrared light, memorize a phone book, or cause others to move by willing them to with our own mind? Would we be human if we ceded the role of smartest beings on earth to others, or would those enhanced beings be humanity's new form? Would we be human if our minds never operated independently from others'? If they operated for eternity? These questions raise the more general question of what it means to be human, and also the question of whether being human by any particular definition has intrinsic value. Although these questions are age-old, they arise with a new urgency in connection with advanced BCIs (McGee and Maguire, 2007).

BCIs could eventually bring about tectonic societal shifts. The effects of cochlear implants on deaf culture (Tucker, 1998) may presage the impact of BCIs on all aspects of human culture that are adapted to current human capabilities rather than the augmented, networked capabilities of future cyborgs. Global political trends such as the Arab Spring, attributed in part to social networking, could be distant harbingers of the effect of wide bandwidth BCIs that enable the sharing of thoughts and feelings among groups of individuals.

### Medium-term challenges

The ethical challenges of the medium term will be largely free of the existential and metaphysical dimensions just discussed, such as ceding dominion of the earth to others, merging our identities into a global network, or questioning the meaning and value of being human. Yet the emergence of these medium-term challenges will depend on BCI technology advancing significantly beyond its current state and therefore do still have a science fiction flavor. We take the defining feature of the medium-term to be the availability of BCI therapies for a wide range of human afflictions.

One set of ethical issues at this point will concern barriers to delivery of BCI therapies. One barrier is likely to be economic. The cost of BCIs in the coming decades is hard to predict, as is future societal support for universal access, but we can anticipate some degree of inequity in access to BCIs. Patient acceptance of implants, in particular the bioethicists' “yuck factor” (Niemelä, 2011) may also prevent or slow delivery of BCI therapies.

Another issue with a degree of bioethical precedent concerns control of the BCI (Jebari and Hansson, 2013). Therapies vary in how much control patients have. How much adjustment or programming of a BCI by the patient will be feasible, and with what risks and benefits? An aspect of control with much less precedent concerns the possibility of BCI hacking, that is, the possibility that a third party, unbeknownst to patient and healthcare provider, would deliver inputs to a patient's brain or read brain states (Denning et al., 2009). Although safeguards against such interventions could of course be designed into any system, experience with current information technology shows that no system can ever be made entirely invulnerable to such attacks. This raises the dire prospect of hacking into systems controlling human thought, feeling and behavior.

Finally, as BCIs are adopted more widely for therapeutic use, enhancement uses will likely follow. The ethics of enhancement is a well-explored area in bioethics and neuroethics more specifically (Parens, 2000; Farah et al., 2014). However, compared to the effects of drugs, which only modulate function within existing brain networks, the addition of new sensory, action and computational devices has the potential to more radically enhance human capabilities.

The transition from therapy to enhancement is evident in many areas of medicine: from neuropsychiatry, where attention-boosting drugs are used by college students to enhance their focus and improve their grades (Sussman et al., 2006), to plastic surgery, where techniques developed to reconstruct injured faces are now commonly used to enhance the attractiveness of the uninjured. Therapeutic implantation of BCIs for only mildly impaired individuals could provide a bridge between therapy and enhancement uses of BCI. Another pathway along which BCIs could transition from therapy to enhancement is by the addition of enhancing BCIs for someone who is already undergoing therapeutic implantation for a serious disorder, for example increasing the range of wavelengths perceived by someone with impaired vision.

### Near-term challenges

Compared with the challenges awaiting us in the medium and long-term, which are currently speculative, we are now faced with a set of ethical challenges that are clear and present. How we respond to these challenges will determine the path we take to the medium- and long-term challenges of BCIs. One set of ethical challenges concerns clinical trials—how trials are conducted and which indications are pursued. Economic considerations of course shape these decisions, and the ethical issue is how these considerations will be balanced by other considerations. Will BCIs be developed only for the most profitable applications, leaving numerous “orphan” medical conditions unaddressed? How will an emphasis on profitability shape the future of BCI technology, by advancing technology for certain applications over others?

A related set of immediate challenges concerns ownership of products during research and development. The finances of pharmaceutical research and development has been identified as an impediment to discovery and innovation (Cohen, 2005), as well as a disincentive for scientific objectivity and transparency (Goldacre, 2013). Given the extensive collaboration of academic and other publicly funded researchers with privately held device manufacturers, how should we design intellectual property agreements, clinical trials and regulation to maximize innovation and patient benefit?

## Approaching the future step by step

We cannot help but approach the future step by step. In contrast, ethical analyses sometimes jump ahead. This is motivated by the commendable desire to anticipate, and therefore more effectively address, the ethical issues of the future. But this long-term ethical forecasting can be counterproductive. One reason is that it takes our attention away from the vitally important, if more mundane, ethical issues of today. Pondering what it means to be human may be more interesting than projecting research and development output under different intellectual property regimes. However, there is only one opportunity to get current decisions right, and that is now. Another reason is that bioethical decisions depend not only on values and principles, but also on the facts of the matter, which we have limited ability to anticipate in the distant future. Only as we live through each of the eras summarized in Table 1 will we be positioned to predict the likely empirical constraints on the ethical decisions of the next era.

**Table 1:** **Three eras of BCIs and their corresponding ethical challenges**.

**Time period**	**Characteristics**	**Ethical challenges**
Long-term	Extensive enhancement of human brains with BCIs	Transhuman cyborgs treatment of humans as inferior and inconsequential
		Vulnerability of those with BCIs to thought control and mind-reading
		Loss of individuality to a merged group mind
		Immortality of thoughts, memories and whole minds
Medium-term	Routine use of BCIs as therapy	Cost of BCIs as an obstacle for needy patients
		Discomfort or disgust with implants as an obstacle for patients
		Question of who controls operation of patients' BCIs
		Security of BCIs against hacking
		Acceptance of BCIs for enhancement of normal function
Near-term	Use of BCIs in a translational research setting	Conduct of clinical trials
		Developing BCI systems that maximize benefit as opposed to profit
		Ownership of intellectual property as an impediment or incentive to biomedical advances
		The influence of funding sources on research priorities

For example, how current BCI work is financed, who controls intellectual property and so forth, will set our course toward the next set of issues, the decisions on what gets commercially developed, how patients get access, and what kind of enhancements are available. Then, depending on our experience with whatever mix of technologies is developed in that medium-term period—which sensory, motor and cognitive systems can be effectively augmented for therapy and enhancement, what unanticipated benefits and harms emerge—we will have a much better platform from which to foresee and address the currently imponderable long-term ethical challenges of BCIs and transhumanism.

### Conflict of interest statement

The authors declare that the research was conducted in the absence of any commercial or financial relationships that could be construed as a potential conflict of interest.

## References

[B1] Berger T. W. Hampson R. E. Song D. Goonawardena A. Marmarelis V. Z. Deadwyler S. A. (2011). A cortical neural prosthesis for restoring and enhancing memory. J. Neural. Eng. 8:046017 10.1088/1741-2560/8/4/046017 21677369 PMC3141091

[B2] Cohen F. J. (2005). Macro trends in pharmaceutical innovation. Nat. Rev. Drug. Discov. 4, 78–84 10.1038/nrd1610 15688075

[B3] Denning T. Matsuoka Y. Kohno T. (2009). Neurosecurity: security and privacy for neural devices. Neurosurg. Focus 27:E7 10.3171/2009.4.FOCUS0985 19569895

[B4] Farah M. J. Smith M. E. Ilieva I. Hamilton R. H. (2014). Cognitive enhancement. Wiley Int. Rev. Cogn. Sci. 5, 95–103 10.1002/wcs.1250 26304298

[B5] Goldacre B. (2013). Bad Pharma: How Drug Companies Mislead Doctors and Harm Patients. New York, NY: Random House LLC

[B6] Jebari K. Hansson S.-O. (2013). European public deliberation on brain machine interface technology: five convergence seminars. Sci. Eng. Ethics 19, 1071–1086 10.1007/s11948-012-9425-0 23263902

[B7] Klauke S. Goertz M. Rein S. Hoehl D. Thomas U. Eckhorn R. (2011). Stimulation with a wireless intraocular epiretinal implant elicits visual percepts in blind humans. Invest. Ophthalmol. Vis. Sci. 52, 449–455 10.1167/iovs.09-4410 20861492

[B8] Lebedev M. (2014). Brain-machine interfaces: an overview. Trans. Neurosci. 5, 99–110 10.2478/s13380-014-0212-z

[B9] Lipsman N. Zener R. Bernstein M. (2009). Personal identity, enhancement and neurosurgery: a qualitative study in applied neuroethics. Bioethics 23, 375–383 10.1111/j.1467-8519.2009.01729.x 19527265

[B10] McGee E. M. Maguire G. Q. (2007). Becoming borg to become immortal: regulating brain implant technologies. Camb. Q. Healthc. Ethics 16, 291–302 10.1017/S0963180107070326 17695620

[B11] Mori M. (1970). The uncanny valley. Energy 7, 33–35 10.1093/scan/nsr025 21515639 PMC3324571

[B12] Niemelä J. (2011). What Puts the ‘Yuck’ in the Yuck Factor?. Bioethics 25, 267–279 10.1111/j.1467-8519.2010.01802.x 20184561

[B13] Niparko J. K. Tobey E. A. Thal D. J. Eisenberg L. S. Wang N.-Y. Quittner A. L. (2010). Spoken language development in children following cochlear implantation. JAMA 303, 1498–1506 10.1001/jama.2010.451 20407059 PMC3073449

[B14] Parens E. (Ed.). (2000). Enhancing Human Traits: Ethical and Social Implications. Washington, DC: Georgetown University Press.

[B15] Sussman S. Pentz M. A. Spruijt-Metz D. Miller T. (2006). Misuse of “study drugs:” prevalence, consequences, and implications for policy. Subst. Abuse Treat. Prev. Policy 1:15 10.1186/1747-597X-1-15 16764722 PMC1524735

[B16] Torab K. Davis T. S. Warren D. J. House P. A. Normann R. A. Greger B. (2011). Multiple factors may influence the performance of a visual prosthesis based on intracortical microstimulation: nonhuman primate behavioural experimentation. J. Neural. Eng. 8:035001 10.1088/1741-2560/8/3/035001 21593550 PMC3144033

[B17] Tucker B. P. (1998). Deaf culture, cochlear implants, and elective disability. Hast. Cent. Rep. 28:6 10.2307/3528607 9762533

[B18] Velliste M. Perel S. Spalding M. C. Whitford A. S. Schwartz A. B. (2008). Cortical control of a prosthetic arm for self-feeding. Nature 453, 1098–1101 10.1038/nature06996 18509337

[B19] Warwick K. (2003). Cyborg morals, cyborg values, cyborg ethics - Springer. Ethic. Inf. Technol. 5, 131–137

